# Ultrasound elastography: compression elastography and shear-wave elastography in the assessment of tendon injury

**DOI:** 10.1007/s13244-018-0642-1

**Published:** 2018-08-17

**Authors:** Rui Prado-Costa, João Rebelo, João Monteiro-Barroso, Ana Sofia Preto

**Affiliations:** 1 Department of Physical and Rehabilitation Medicine, Centro Hospitalar São João, Alameda Prof. Hernâni Monteiro, 4200-319 Porto, Portugal; 2 Department of Radiology, Centro Hospitalar São João, Porto, Portugal

**Keywords:** Elastography, Sonoelastography, Ultrasound, Tendon injury, Tendinopathy

## Abstract

**Abstract:**

Ultrasound elastography (USE) is a recent technology that has experienced major developments in the past two decades. The assessment of the main mechanical properties of tissues can be made with this technology by characterisation of their response to stress. This article reviews the two major techniques used in musculoskeletal elastography, compression elastography (CE) and shear-wave elastography (SWE), and evaluates the studies published on major electronic databases that use both techniques in the context of tendon pathology. CE accounts for more studies than SWE. The mechanical properties of tendons, particularly their stiffness, may be altered in the presence of tendon injury. CE and SWE have already been used for the assessment of Achilles tendons, patellar tendon, quadriceps tendon, epicondylar tendons and rotator cuff tendons and muscles. Achilles tendinopathy is the most studied tendon injury with USE, including the postoperative period after surgical repair of Achilles rupture tendon. In relation to conventional ultrasound (US), USE potentially increases the sensitivity and diagnostic accuracy in tendinopathy, and can detect pathological changes before they are visible in conventional US imaging. Several technical limitations are recognised, and standardisation is necessary to ensure repeatability and comparability of the results when using these techniques. Still, USE is a promising technique under development and may be used not only to promote an early diagnosis, but also to identify the risk of injury and to support the evaluation of rehabilitation interventions.

**Key Points:**

• *USE is used for the assessment of the mechanical properties of tissues, including the tendons.*

• *USE increases diagnostic performance when coupled to conventional US imaging modalities.*

• *USE will be useful in early diagnosis, tracking outcomes and monitoring treatments of tendon injury.*

• *Technical issues and lack of standardisation limits USE use in the assessment of tendon injury.*

## Introduction

Ultrasound (US)-based methods are of particular interest due to their inherent advantages, such as wide availability, relatively low cost and quick procedures [1]. Over the last two decades, there has been significant development in different methods to perform tissue elasticity measurements [2]. Some authors claim that ultrasound elastography (USE) could represent the most important advance in US medical imaging since Doppler establishment [3].

USE can be applied in a variety of medical fields. In oncology, it can be used to assess liver, breast or prostatic lesions. Evaluation of the thyroid gland, gynaecological or musculoskeletal pathologies are also possible using USE [4].

USE is based on the principle that the application of a stress force on a tissue will induce internal displacements intrinsically related to its elastic properties [5, 6].

There are different techniques of USE which share three common steps: excitation application (stress), tissue response measurement (strain) and mechanical parameters estimation [7].

Applying a focused radiation force from a linear US array which induces shear waves defines shear-wave elastography (SWE) [8]. The repeated manual compression of tissues by using a hand-held US transducer to produce strain defines compression elastography (CE) or static strain elastography [9].

The elastic modulus is a parameter used to quantify elasticity. The elastic modulus has larger variation compared to the parameters of other imaging modalities, allowing higher discrimination between different tissues and between normal and pathologic tissues [7]. This elastic modulus can be defined as the slope of a stress–strain curve, during elastic strain, reflecting the elastic properties of the tissues, and it is inversely proportional to the degree of strain, assuming that applied stress is uniform [10]. The Young’s elastic modulus (E) and shear elastic modulus (G) represent the elastic modulus and are most applicable to biological tissues.

US is an exceptional diagnostic tool for tendon injuries evaluation, including tendinopathies and tendon ruptures [11]. The biomechanical modifications arising from physiological and pathologic processes can be assessed by using USE, which has a great applicability on early diagnosis [9].

Tendinopathy is defined as an overuse injury histologically characterised by the proliferation of tenocytes, collagen fibre disorganisation, increase of non-collagenic matrix, fluid accumulation between fibres, capillary proliferation and calcification, which can all induce modifications of the viscoelastic properties of the tendon and, consequently, be noticeable on USE [12–14]. Early diagnosis of tendinopathies is crucial to implement conservative measures to avoid severe tendinous injury or even rupture. The modifications on tissue elasticity can be early detected by USE potentially even before there are visible abnormalities on conventional B-mode ultrasonography [15, 16]. USE may also play a role on rehabilitation, as a tool to monitor and guide ongoing treatments, including after a surgical repair of a tendon rupture, and to predict the return to previous activities (especially in high-level athletes) [17, 18].

This article intends to review the two major techniques used in USE, CE and SWE, and the studies published on major electronic databases that use both techniques in tendon pathology evaluation. The major findings regarding elasticity characterisation, correlations, performance characterisation and current limitations of this technology are featured in the present review.

### Compression elastography (CE)

**Fig. 1:**
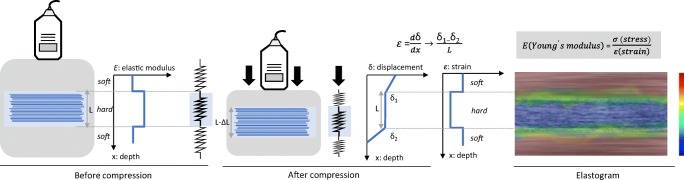
Basic physical principle of compression elastography (CE). The operator exerts compression on the tissue, generating a displacement (δ) that can be estimated by comparing the structure at rest and under compression. The strain (ε) is the ratio difference in displacement between two points to their distance pre-compression (L). The strain measurements are displayed as a colour map, called an elastogram, which is overlaid on the B-mode image. Typically, low strain (stiff tissue) is displayed in blue and high strain (soft tissue) is displayed in red

CE is a qualitative or semi-quantitative technique based on the application of compressive waves on tissues [3]. During examination, the operator executes rhythmic and regular compressions in the area of interest to obtain an axial strain on the tissue. Given a certain amount of applied stress, softer tissues have more deformation and, therefore, experience larger strain than stiffer tissues. These strains are obtained by the variation of longitudinal distance, calculated from the time taken by the US waves to return to the transducer before and after compression (Fig. 1). A specialised software encodes the signals obtained and then displays on the US screen a colour-coded elastogram. For the colour elastogram, red usually indicates soft consistency, blue indicates hard consistency, and green and yellow encode intermediate stiffness [19].

Young’s elastic modulus (E) is inversely proportional to the measured strain (*E* = stress/strain) based on Hooke’s law [2]. The determination of the Young’s modulus requires quantification of the stress distribution within the tissue, which is challenging using CE because the applied force (stress) can be highly variable and not standardised. This is a major drawback as, unlike Young’s modulus values, the data acquired by this technique do not represent tissue intrinsic properties. However, there are semi-quantitative methods that can be used, such as the strain ratio, an index of the relative elasticity between a chosen region of interest (ROI) in the examined tissue and a reference ROI, usually the adjacent subcutaneous tissues. A visual scoring system that compares the colour of the compressed area to the colour of the surrounding area could also be used [20].

CE is also affected by the depth of the tissues of interest and probe position, increasing the difficulty in obtaining precise measurements with this highly operator-dependent procedure [21].

### Shear-wave elastography (SWE)

In addition to morphological information, SWE can also quantify the absolute elasticity value of soft tissue structures [22].

**Fig. 2:**
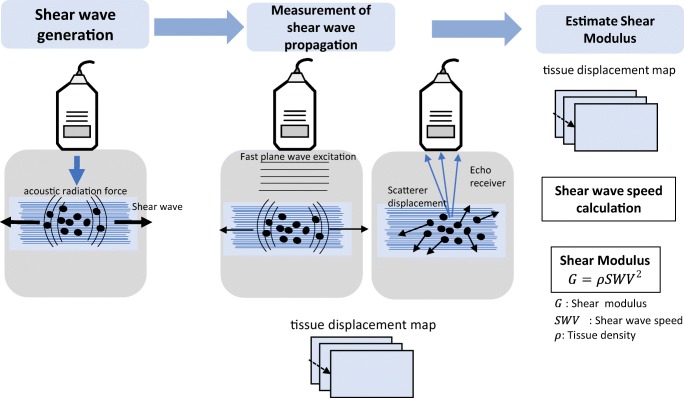
Basic physical principle of shear-wave elastography (SWE). A perpendicular stress force (acoustic radiation force) is applied to the tissue, which causes the generation of shear waves. The velocity of the shear wave could be measured by obtaining radiofrequency images with a high frame rate, which can be used to generate a tissue displacement map. Tissue displacements are used to calculate the shear-wave velocity (V_s_) and shear modulus (G)

Shear waves can be induced through various methods, such as US push beams (supersonic shear imaging) or by external mechanical vibrations (transient elastography). These waves can be either longitudinal, where the particles oscillate in the direction of wave propagation, or transversal, in which particles oscillate perpendicular to the direction of wave propagation. The transverse wave propagation speed is called the shear-wave velocity (V_s_) (Fig. 2) [23].

Transient elastography uses an external actuator to provide a single cycle of low-frequency vibration that generates shear waves and US methods to track the resulting motion. Motion estimation can be performed using cross-correlation of consecutively acquired radiofrequency data. It measures only regional elasticity with limited depth and provides only a relative measure of stiffness [2].

Supersonic shear imaging creates shear waves over a greater range of depth by using multiple push beams focused at different depths within the tissue and then uses ultra-high frame rate ultrasonic imaging to track these waves as they propagate [23].

The obtained tissue displacement maps are used to calculate the propagation velocity of the shear waves. The distribution of shear-wave velocities is directly related to the shear modulus G, with the latter being defined as $G=\rho {V}_s^2$ where *ρ* is the material density. The shear modulus is sensitive to the probe positioning and shear waves have the potential for faster propagation along the longitudinal axis of the tendon [24]. The shear modulus discloses good correlation with the Young’s elastic modulus (E) and can be related by the following equation: $E\cong 3G=3\rho {V}_s^2$ [23]. The more isotropic and homogeneous the tissue, the more accurate the estimate of the elasticity modulus. This determination is not accurate for anisotropic and heterogeneous tissues like tendons that combine elastic and viscous properties. Nevertheless, the elastic modulus can be used as an objective indicator of the relative elasticity of tissues of the same type under the same testing conditions [15, 25].

SWE allows the assessment of qualitative elastograms and quantitative measurements, and it is considered more objective, since it is less influenced by inter-operator variability, providing potentially more reproducible results than CE. However, some limitations must be considered, particularly the limited size, shape and depth of the ROI [26].

## Materials and methods

A bibliographic research was made on PubMed, Ovid MEDLINE, Cochrane Library, EMBASE and PEDro for articles published up to February 2018 related to the assessment of pathologic tendons, ruptured tendons, tendons with tendinopathy and on systemic diseases, using CE or SWE techniques. The keywords “elastography”, “US”, “sonoelastography”, “shear wave”, “tendinopathy”, “tendon”, “tendons”, “real-time” and “strain” were used in different combinations.

## Results

A total of 39 studies assessing pathologic tendons were found using USE, 26 using CE (Table 1) and 13 using SWE (Table 2).

**Table 1:** Studies found using compression elastography (CE) in the assessment of injured or pathologic tendons

Authors year [reference]	Subjects (symptomatic and asymptomatic; structures assessed)	Study design, comparison modality	Major findings	Diagnostic performance and correlations (including comparison with clinical examination, functional scores, conventional US and MRI)
Achilles tendon				
De Zordo et al. 2010 [27]	25 volunteers/50 asymptomatic tendons. 25 patients/25 tendons.	Cross-sectional, case–control study. Comparison of USE with conventional US using clinical examination as reference standard.	Symptomatic tendons were softer. The distal and middle third were more frequently involved than the proximal third of Achilles tendon.	USE detected more alterations in contralateral asymptomatic tendons than conventional US. USE has comparable accuracy to conventional US and clinical examination. USE showed good correlation with conventional US (*κ* = 0.89, *p* < 0.001), being greater for lesions of the distal third compared to the middle and proximal parts. Ac: 97%, Sp: 99.2%, Sv: 93.7% compared to standard clinical findings. Ac: 99%, Sp: 100%, Sv: 93% compared to conventional US.
Sconfienza et al. 2010 [28]	18 volunteers/36 asymptomatic tendons. 12 patients/12 tendons.	Cross-sectional, case–control study. Comparison of USE with conventional US/MRI using clinical symptoms as reference standard.	Symptomatic tendons were harder. No difference between symptomatic and control tendons at the enthesis and myotendinous junction.	USE showed good correlation with conventional US and MRI.
Tan et al. 2012 [29]	20 volunteers/40 asymptomatic tendons. 16 patients in post-operative period for tendon rupture correction/19 tendons.	Cross-sectional, case–control study.	Healing tendons demonstrated a harder and heterogeneous texture compared to the healthy ones.	
Gehmert et al. 2012 [30]	9 rabbits: 12 ruptured tendons, 6 tendons treated with stem cells, 6 ruptured tendons not treated, 6 healthy tendons.	Laboratory study with New Zealand white rabbits.	Treated tendons with stem cells had higher elasticity compared with those not treated. Stem cells restored the elastic properties of Achilles tendon.	
Klauser et al. 2013 [31]	10 cadavers/13 tendons.	Laboratory study using cadaveric models. Comparison with conventional US using clinical histology as reference standard.	Injured tendons were softer than asymptomatic tendons.	USE showed perfect agreement with histology results and moderate agreement with conventional US (*κ* = 0.52, *p* < 0.001). Sv: 100%, Sp: 100% (histologic findings as the reference standard).
Turan et al. 2013 [32]	41 patients with ankylosing spondylitis (AE)/82 tendons. 32 healthy volunteers/64 tendons.	Cross-sectional, case–control study. Comparison with conventional US using clinical examination as reference standard.	Distal third of Achilles tendon was softer on patients with AE compared with healthy individuals (*p* = 0.001) and it was associated with enthesopathy findings (*p* = 0.07). The intensity of achillodynia tended to be higher in patients with abnormal USE examination findings (*p* = 0.07).	USE had moderate to good correlation with conventional US (*κ* = 0.80 for proximal third, *κ* = 0.58 for middle third, *κ* = 0.39 for distal third).
Evranos et al. 2015 [33]	78 patients with diabetes: 35 patients with foot ulcers, 43 patients without foot ulcers, 33 healthy individuals	Cross-sectional, case–control study.	The Achilles tendon was softer in diabetic patients with foot ulcers than in patients without ulcers (*p* < 0.001) or in non-diabetics (*p* = 0.03). A softer medial third of the Achilles tendon was related to longer duration of diabetes, use of insulin, foot ulcers and presence of neuropathy or peripheral arterial disease.	
Ooi et al. 2015 [34]	120 patients/120 symptomatic tendons 120 volunteers/120 asymptomatic tendons	Cross-sectional, case–control study. Comparison with conventional US and functional scores using clinical examination as reference standard.	Symptomatic tendons were softer than asymptomatic tendons (SR = 1.70 ± 0.84 vs. 0.76 ± 0.30, *p* < 0.001).	USE had excellent sensitivity, specificity and diagnostic accuracy (Sv: 97.5%, Sp: 94.5%, Ac: 97.8%). USE had excellent correlation with clinical findings (*k* = 0.91, *p* < 0.05). USE showed good to excellent agreement with B-mode US (*k* = 0.81, *p* < 0.001). SR had moderate correlation with functional scores (*ρ* = − 0.62, *p* < 0.001).
Busilacchi et al. 2016 [17]	30 volunteers/60 asymptomatic tendons. 25 patients in post-operative period of tendon rupture correction/50 tendons.	Prospective cohort study. Comparison of USE with functional score.	After surgery, the tendons were harder and achieved a peak thickness 6 months after, compared to contralateral and control group tendons. Contralateral tendons were stiffer than control group tendons (*p* < 0.001).	SR had a negative correlation with functional score (*ρ* = − 0.42, *p* = 0.03).
Onal et al. 2016 [35]	42 acromegaly patients/84 tendons. 42 healthy volunteers/84 tendons.	Cross-sectional, case–control study. Comparison of USE with biological markers.	Achilles tendons in patients with acromegaly were softer than the healthy ones (*p* = 0.0001).	No significant correlation between USE and biological markers of the disease.
Yamamoto et al. 2017 [36]	24 New Zealand rabbits with Achilles tendon transection.	Laboratory study. SR calculation and mechanical testing of ultimate load, ultimate stress, elastic modulus and linear stiffness and histological analysis at weeks 2, 4, 8 and 12.	Mean SR at the healing site after Achilles tendon rupture gradually decreased and the Achilles tendon became significantly harder over time (*p* < 0.001).	SR showed correlation with all mechanical evaluations [ultimate stress (*ρ* = 0.68, *p* < 0.001), elastic modulus (*ρ* = 0.78, *p* < 0.001) and histologic evaluation of the healing site (*ρ* = 0.87, *p* < 0.001)].
Patellar tendon				
Rist and Mauch 2012 [37]	75 athletes: 37 asymptomatic tendons, 38 symptomatic tendons.	Cross-sectional, case–control study.	Symptomatic tendons showed higher strain scores than asymptomatic ones in longitudinal and cross-section.	
Ooi et al. 2016 [38]	35 volleyball athletes: 40 symptomatic tendons, 30 asymptomatic tendons.	Cross-sectional, case–control study. Comparison with conventional US using clinical examination as reference standard.	Symptomatic tendons were softer than asymptomatic tendons.	USE alone: Ac: 62.9%, Sv: 70%; Sp: 53.3%. USE + B-mode US: Ac: 61.4%, Sv: 82.5%, Sp: 33.3%. USE + power Doppler US: Ac: 60.0%, Sv: 72.5%, Sp: 43.3%. Specificity of USE alone and combined with conventional US were low. USE may increase conventional US sensitivity and accuracy in the diagnosis of patellar tendinopathy. Softening of symptomatic patellar tendons were better related with functional scores (*p* = 0.004) than conventional US (*p* = 0.10).
Epicondylar tendons				
De Zordo et al. 2009 [39]	28 volunteers/44 asymptomatic tendons. 32 patients with lateral epicondylitis/38 symptomatic tendons.	Cross-sectional, case–control study. Comparison with conventional US using clinical examination as reference standard.	Symptomatic tendons were softer than asymptomatic tendons (*p* < 0.001).	Sv: 100%, Sp: 89%, Ac: 94%. USE had higher sensitivity and accuracy than conventional US in detecting clinically symptomatic epicondylar tendinopathy. USE had higher sensitivity than conventional US in detecting intratendinous and peritendinous lesions. Good correlation with conventional US findings (*ρ* ≥ 0.900, *p* < 0.001).
Ahn et al. 2014 [40]	79 patients and 14 healthy volunteers. 97 symptomatic tendons. 89 asymptomatic tendons.	Cross-sectional, case–control study. Comparison with conventional US and clinical parameters using clinical examination as reference standard.	Symptomatic tendons were softer (mean SR = 1.45 ± 0.45) than asymptomatic tendons (mean SR = 2.07 ± 0.70) (*p* < 0.001).	Significant correlation with conventional US (*p* < 0.001).
Park et al. 2014 [41]	28 patients with unilateral lateral epicondylitis: 14 symptomatic tendons, 14 asymptomatic tendons.	Cross-sectional, case–control study. Comparison with conventional US using clinical examination as reference standard.	Symptomatic tendons were softer than asymptomatic tendons.	USE alone: Sv: 96.4%, Sp: 96.4%, Ac: 96.4% (*p* < 0.01). USE had greater diagnostic accuracy compared to conventional echography (Ac: 96.4% vs. 89.5%, *p* < 0.01). USE was correlated with severity of tendon pathology estimated by conventional US (*p* < 0.01). USE was correlated with cortical irregularities around the lateral epicondyle, history of steroid injection and symptom duration (*p* < 0.05). USE findings had correlation with pain during resistive middle finger extension on physical examination (*p* < 0.05).
Kocyigit et al. 2016 [42]	17 volunteers with lateral epicondylitis/34 tendons.	Cross-sectional, case–control study. Comparison with conventional US using clinical examination as reference standard.	Symptomatic tendons were softer than asymptomatic tendons (*p* < 0.001). Mean SR: medial portion = 0.45 ± 0.12, middle portion = 0.44 ± 0.8, lateral portion = 0.47 ± 0.19.	USE was superior to conventional US distinguishing healthy tendons from those with tendinopathy. The SR of medial portion of the extensor tendon correlates with night pain (*ρ* = − 0.522, *p* = 0.03) and duration of symptoms (*ρ* = − 0.61, *p* = 0.01).
Klauser et al. 2017 [43]	16 cadavers/25 common flexor tendons.	Laboratory study using cadaveric models. Agreement comparison of conventional US and USE with histological evaluation.	Tendons with tendinopathy were softer than normal tendons.	USE combined with conventional US had better correlation (*k* = 0.84) with histology compared with the use of B-mode US (*k* = 0.57) or USE alone (*k* = 0.68) (*p* < 0.02).
Klauser et al. 2017 [44]	17 cadavers/26 common flexor tendons.	Laboratory study using cadaveric models. Agreement comparison of conventional US and USE with histological evaluation.	Tendons with tendinopathy were softer than normal tendons.	USE alone: Sv: 85%, Sp: 86%, Ac: 86%. USE + B-mode US: Sv: 95%, Sp: 81%, Ac: 92%. USE in combination with conventional US provided improved sensitivity (*p* < 0.02) without loss of specificity and had correlation with histology evaluation (*κ* = 0.78, *p* < 0.02).
Rotator cuff tendons				
Seo et al. 2014 [45]	98 patients/101 shoulders with lesions (cuff tears, tendinopathies, adhesive capsulitis, calcific tendinitis, labral lesions).	Cross-sectional. Comparison of USE with conventional US/MRI using MRI findings as reference standard.	USE allowed quantification of the severity of adipose involution on the supraspinatus muscle tendon.	Sv: 95.6%, Sp: 87.5%, Ac: 91.1%. USE had excellent correlation with MRI (*ρ* = 0.855, *p* < 0.001) and conventional US (*ρ* = 0.793, *p* < 0.001).
Seo et al. 2014 [46]	118 patients/118 shoulders with supraspinatus tendinopathy.	Cross-sectional. Comparison of USE with conventional US/MRI using MRI findings as reference standard.	Tendons with tendinopathy had focal areas of softness.	Positive correlation between grades of MRI and USE (*ρ* = 0.829, *p* = < 0.001). Positive correlation between grades of US and USE (*ρ* = 0.723, *p* = < 0.001).
Tudisco et al. 2015 [47]	100 shoulders: 50 with supraspinatus tear, 50 healthy contralateral shoulders.	Cross-sectional, case–control study. Comparison of USE (SR) of the tendons between the two groups. Comparison of USE (SR) with demographic data and functional scores.	Mean SR in the affected shoulder (0.75 ± 0.08) was lower than the contralateral healthy shoulder (1.01 ± 0.07) (*p* < 0.0001).	Negative correlation between SR and VAS (Visual Analogue Scale) score for pain (*r* = − 0.76). Strong positive correlation between SR and functional scores.
Kocyigit et al. 2016 [48]	50 shoulders: 25 patients diagnosed with unilateral subacromial impingement, 25 healthy shoulders.	Cross-sectional, case–control study. Comparison of USE (including SR) of the tendons between the two groups. Comparison of SR of the tendons with demographic data and functional scores.	Decreased stiffness of the supraspinatus tendon of the affected shoulder compared to healthy shoulder (*p* < 0.001).	There was no correlation between the findings in USE and functional scores, gender and age.
Lee et al. 2016 [49]	39 patients with chronic supraspinatus tendinopathy.	Cross-sectional. Comparison of the supraspinatus tendon SR with the degree of tendinosis on MRI.		Positive correlation of SR with degree of tendinosis in MRI (*p* < 0.001).
Long head of biceps tendon (LHBT)				
Seo et al. 2014 [50]	34 patients/38 shoulders with tendinopathy of LHBT. 98 patients/114 shoulders without tendinopathy of LHBT.	Cross-sectional, case–control study. Comparison of USE between the two groups. Comparison with conventional US using clinical examination as reference standard.	Focal areas of softening in affected tendons.	Positive correlation between USE and conventional ultrasound (*ρ* = 0.585, *p* < 0.001).
Quadriceps tendon				
Teber et al. 2015 [51]	53 patients with chronic renal failure undergoing dialysis/106 quadriceps tendons. 25 healthy individuals/50 quadriceps tendons.	Cross-sectional, case–control study. Comparison of USE of the quadriceps tendon between the two groups.	Quadriceps tendons in patients with chronic renal failure were softer (right knee, *p* = 0.03; left knee, *p* = 0.02) compared to controls.	

*Ac* accuracy; *MRI* magnetic resonance imaging; *Sp* specificity; *Sv* sensitivity; *SR* strain ratio; *ρ* Spearman rank correlation coefficient; *r* Pearson correlation coefficient; *k* kappa coefficient

**Table 2:** Studies found for shear-wave elastography (SWE) in the assessment of injured or pathologic tendons

Authorsyear [reference]	Subjects (symptomatic and asymptomatic; structures assessed)	Study design, comparison modality	Major findings	Diagnostic performance and correlations (including comparison with clinical examination, conventional US and MRI)
Patellar, Achilles and epicondylar tendons				
Dirrichs et al. 2016 [52]	112 patients. Achilles tendon, 41 patients: 34 asymptomatic tendons, 48 symptomatic tendons. Patellar tendon, 38 patients: 25 asymptomatic tendons, 51 symptomatic tendons. Epicondylar tendon, 33 patients: 25 asymptomatic tendons, 41 symptomatic tendons.	Prospective cohort study. Comparison with conventional US using clinical examination as reference standard.	Decreased stiffness in symptomatic tendons. Mean elastic modulus/V_s_ values of symptomatic tendons: 60.3 kPa/4.48 m/s vs. healthy tendons 185 kPa/7.85 m/s (*p* = 0.0004).	Conventional imaging + SWE: Sv 94.3%, Sp: 69.1%, Ac 84.8%. USE increased the sensitivity of conventional US detecting tendinopathy. USE was strongly correlated with clinical symptoms (*ρ* = 0.81, *p* < 0.001).
Coombes et al. 2018 [53]	67 participants: 22 patients with Achilles tendinopathy, 17 patients with patellar tendinopathy, 28 healthy controls.	Cross-sectional, case–control study. Comparison with demographic and functional data.	Achilles tendinopathy: decreased stiffness at the distal insertion (*p* < 0.001). Patellar tendinopathy: increased stiffness in proximal and mid-patellar region (*p* = 0.005).	Achilles tendinopathy: V_s_ < 9.7 m/s, Sp: 81%, Sv: 79%. Patellar tendinopathy: V_s_ > 9.7 m/s, Sp: 82%, Sv: 77%. Lower proximal patellar V_s_ was correlated with age (*r* = − 0.368, *p* = 0.01). Lower Achilles V_s_ was associated with higher age (*r* = − 0.49, *p* < 0.001), greater BMI (*r* = − 0.53, *p* < 0.001), greater pain and disability (*r* = 0.49, *p* < 0.046) and fewer single leg calf raises before pain onset (*r* = 0.646, *p* = 0.001).
Achilles tendon				
Chen et al. 2013 [54]	80 volunteers/36 normal tendons. 14 patients/14 ruptured tendons.	Cross-sectional, case–control study.	Lower and heterogeneous elasticity of torn tendons compared to normal ones, including throughout healing in the subacute stage (*p* = 0.006). Shear modulus of healthy tendons: 291.91 ± 4.38 kPa. Shear modulus of ruptured tendons: 56.48 ± 68.59 kPa.	
Aubry et al. 2015 [12]	80 volunteers/160 asymptomatic tendons. 25 patients/30 symptomatic tendons.	Cross-sectional, case–control study.	Symptomatic tendons were softer (*p* < 0.001). The presence of areas with no signal in the USE images was a sign of tendinous rupture.	Sagittal SWE with tendon in neutral position: V_s_ < 5.7 m/s for tendinopathy diagnosis, Sv 41.7%, Sp 81.8%.
Zhang et al. 2016 [55]	26 volunteers with rupture of the tendon treated surgically.	Prospective cohort study. Comparison to functional scores.	Tendon stiffness had increased over time (*p* < 0.05).	Positive correlation between functional scores and elasticity (*p* = 0.0003).
Patellar tendon				
Zhang et al. 2014 [56]	20 volunteers/40 asymptomatic tendons. 13 patients with unilateral patellar tendinopathy/26 tendons.	Cross-sectional, case–control study. Comparison to functional scores.	Increased stiffness on tendons with tendinopathy (*p* < 0.05).	Significant correlation between increased stiffness in the painful tendon and the intensity of pain and degree of dysfunction.
Rotator cuff tendons				
Lin et al. 2015 [57]	39 patients with calcifying tendinopathy/39 tendons.	Cross-sectional.	USE allowed predicting of calcifications that benefit from fine needle aspiration (*p* < 0.001).	
Rosskopf et al. 2016 [58]	22 asymptomatic volunteers. 44 patients with symptomatic shoulder due to tear or tendinopathy of the supraspinatus tendon.	Prospective cohort study. Comparison of V_s_ of the supraspinatus muscle with MRI characterisation (tendon integrity, tendon retraction, fatty muscle infiltration and muscle volume atrophy).	Mean total V_s_ in tendinopathy of 2.5 ± 0.5 m/s vs. V_s_ of 3.0 ± 0.5 m/s in asymptomatic shoulders (*p* < 0.001). Mean total V_s_ variable according to different grades of tendon retraction (*p* = 0.05). V_s_ decreased with higher fat content and increased in the final stage of fatty infiltration.	
Capalbo et al. 2016 [59]	17 asymptomatic volleyball players/17 upper trapezius on dominant side. 26 volleyball players with rotator cuff tendinopathy/26 upper trapezius on dominant side.	Cross-sectional, case–control study. Comparison of upper trapezius shear modulus between the two groups.	Upper trapezius shear modulus was higher in athletes with rotator cuff tendinopathy than the asymptomatic athletes (*p* = 0.002).	
Hou et al. 2017 [60]	35 patients: 21 symptomatic shoulder assessments, 55 asymptomatic shoulder assessments with B-mode US and SWE.	Retrospective and prospective cohort study. Comparison with morphologic grade of supraspinatus tendon on conventional US.	Proximal supraspinatus tendon (*p* = 0.049) and deltoid (*p* = 0.004) were softer in symptomatic shoulders.	Weak-to-moderate negative correlation between V_s_ of the deltoid muscle and morphologic grade of supraspinatus tendon on conventional US: proximal tendon: *r* = − 0.35; *p* = 0.004; distal tendon: *r* = − 20.32; *p* = 0.007.
Hatta et al. 2017 [61]	45 cadaveric shoulders: 25 shoulders with intact rotator cuff, 20 shoulders with rotator cuff tear.	Laboratory study. Comparison of the shear modulus with the extensibility of the supraspinatus muscle under 30- and 60-N loads.		Moderately significant positive correlation of SWE stiffness with stiffness of the supraspinatus muscle measured by a mechanical device. Significant correlation between the shear modulus of supraspinatus muscle and the experimentally measured extensibility in specimens with intact and torn rotator cuff tendons (*p* < 0.001): 30 N: *r* (intact) = 0.71, *r* (tear) = 0.77; 60 N: *r* (intact) = 0.72, *r* (tear) = 0.78.
Kreplin et al. 2017 [62]	8 patients: 9 shoulders: 5 with full-thickness supraspinatus tendon tear, 2 with partial thickness supraspinatus tendon tear and 1 with tendinosis without tear.	Cross-sectional. Comparison of V_s_ with T2/T2* mapping (MRI) of the supraspinatus tendon.	Average V_s_ = 9.4 ± 2.6 m/s.	Significant negative correlation between T2* and V_s_ (*r* = − 0.86, *p* = 0.013). Significant negative correlation between V_s_ and tear size (*r* range 0.71–0.77, *p* range 0.016–0.034).
Baumer et al. 2018 [63]	19 asymptomatic subjects. 11 patients with full-thickness rotator cuff tear of the supraspinatus tendon.	Retrospective case–control study. Comparison of V_s_ between shoulders with rotator cuff tear and healthy, asymptomatic shoulders. Comparison of V_s_ with age in healthy, asymptomatic shoulders.	Shoulder with supraspinatus tendon tear had lower mean V_s_ in supraspinatus muscle and tendon under active conditions (~ 30° scapular plane active abduction) than healthy, asymptomatic shoulders: mean V_s_ = 3.3 ± 0.8 m/s vs. 4.0 ± 0.4 m/s, *p* = 0.0024. No difference in V_s_ between supraspinatus muscle and tendon under passive conditions.	Sv ≥ 0.67 and Sp ≥ 0.63 to distinguish between asymptomatic control subjects and patients with a rotator cuff tear. Mean V_s_ increased significantly with age in the supraspinatus muscle and tendon of healthy, asymptomatic shoulders (*p* < 0.05, R^2^ ≥ 0.23).

*Ac* accuracy, *BMI* body mass index, *MRI* magnetic resonance imaging, *Sp* specificity, *Sv* sensitivity, *V*_s_ shear-wave velocity, *ρ* Spearman rank correlation coefficient, *r* Pearson correlation coefficient, *R*^2^ coefficient of determination

Almost all studies documented a decrease in tendon stiffness when tendinopathy is present. Conversely, a process of reactive fibrosis can happen in ruptures and, consequently, an increase in tendon stiffness may be observed [18]. Most of the published studies tested CE in tendinopathy; however, in recent years, there has been an increasing interest in SWE as a possible reliable tool to detect and accurately quantify the stiffness of tendons.

### Achilles tendon

The Achilles tendon is the strongest and thickest tendon in the human body, has a superficial location and good differentiation from surrounding tissues, making it particularly suitable for USE assessment [4]. A total of 16 studies (11 using CE and 5 using SWE) were found in the assessment of abnormal Achilles tendon.

Regarding CE, it was demonstrated almost perfect intra-observer repeatability and variable inter-observer repeatability [64–66]. Regarding SWE, it revealed high reproducibility and moderate to high repeatability [64, 67, 68].

**Fig. 3:**
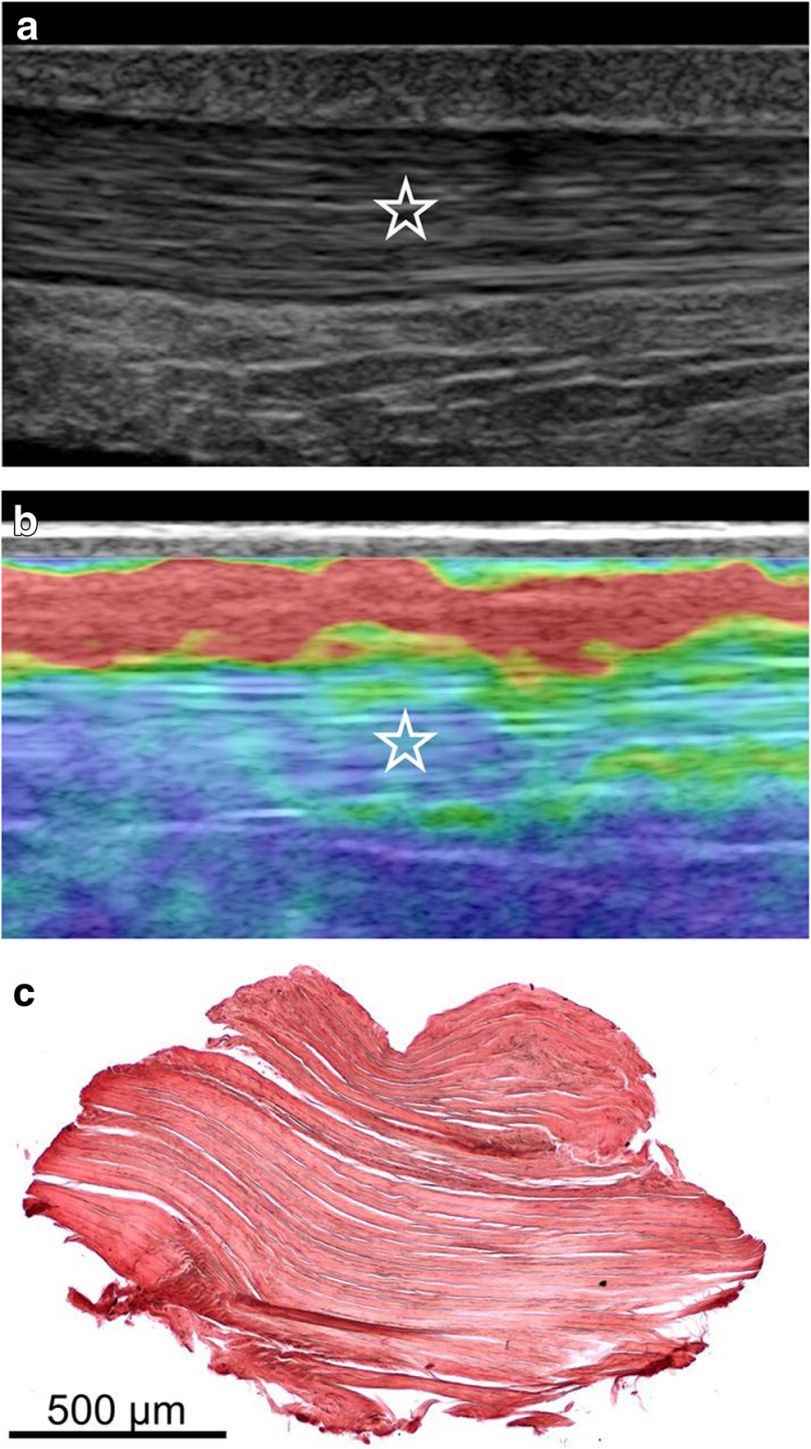
Images of a normal Achilles tendon using CE and histological correlation. **a** Conventional ultrasound (US) image of the middle portion of the Achilles tendon in the longitudinal plane. The star indicates the homogenous fibrillar pattern defining normal tendon appearance. **b** Image of ultrasound elastography (USE) at the same level as in **a**. The blue-green area of ​​the elastogram represented by the star indicates tissue stiffness where biopsy was subsequently performed. **c** Histological image obtained with orcein staining showing parallel collagen fibrils, without adipose infiltration and capillary proliferation. Reproduced, with permission, from Klauser et al. [31], copyright (2013) by the Radiological Society of North America, Inc. (RSNA)

**Fig. 4:**
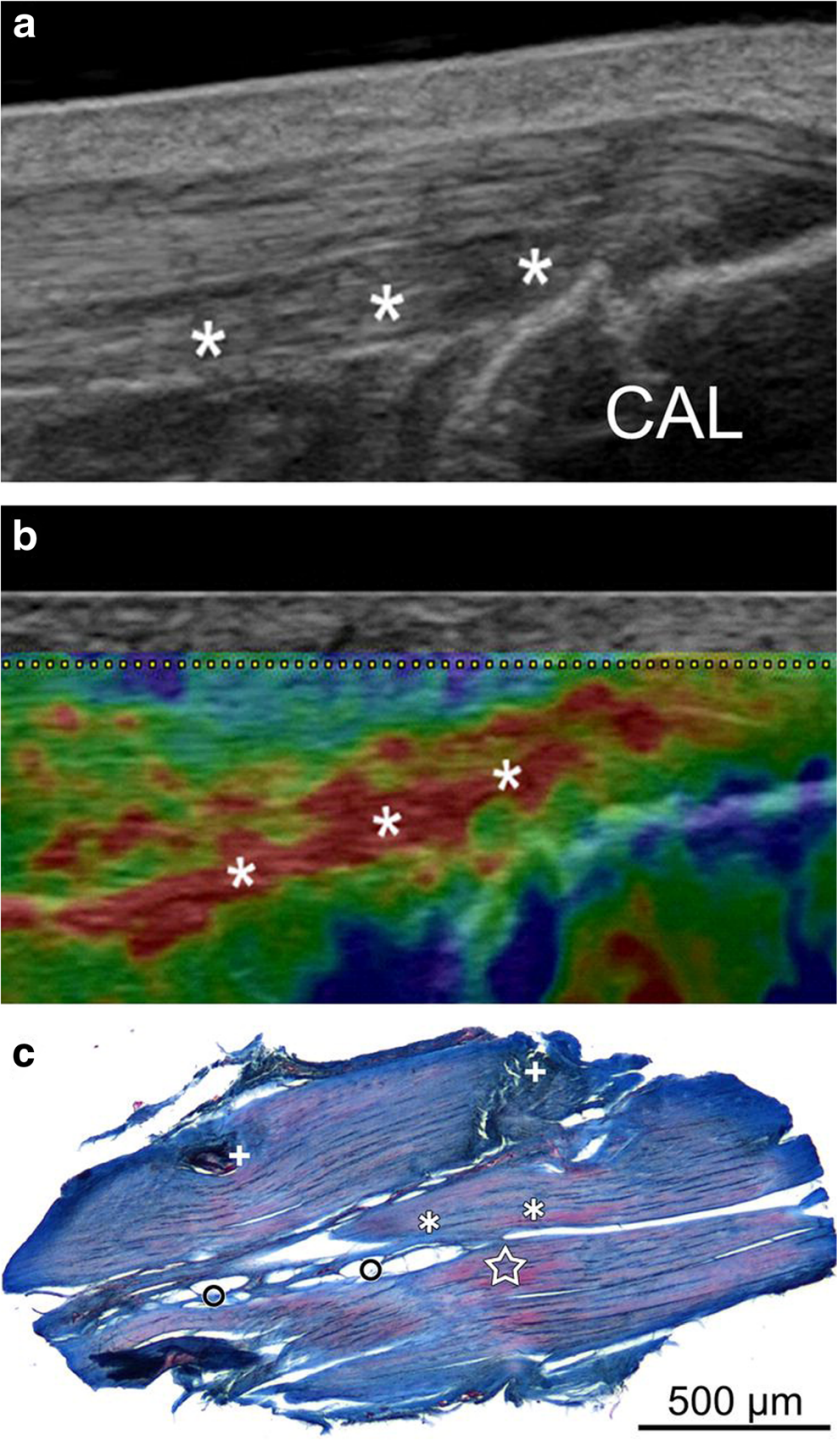
Images of an Achilles tendon with tendinopathy using CE and histological correlation. **a** Conventional US image of the insertion of the Achilles tendon in the longitudinal plane. The asterisks indicate the hypoechoic area without tendon thickening. CAL: calcaneus. **b** Image of USE at the same level as **a**. The red area of ​​the elastogram represented by asterisks is indicative of anomalous decrease of stiffness where biopsy was subsequently performed. **c** Histological image obtained with azan staining showing loss of the parallel fibrillar structure of the collagen, loss of fibre integrity (asterisks), adipose infiltration (circles), capillary proliferation (plus) and mucoid deposition (star). Reproduced, with permission, from Klauser et al. [31], copyright (2013) by the Radiological Society of North America, Inc. (RSNA)

Normal Achilles tendons have a homogenous stiff pattern, whereas pathological ones exhibit a more heterogenous and reduced stiffness pattern [12, 16, 27, 31–34, 52–54], except for one study, using CE, that found symptomatic tendons to be harder than the asymptomatic ones [28]. Studies using CE showed moderate to perfect correlation of USE with conventional US in Achilles tendinopathy evaluation [27, 28, 31, 32] and excellent correlation with functional scores, greater than conventional US [34]. The application of CE demonstrated superior [34] or comparable results [27] in terms of specificity, sensitivity and accuracy compared to conventional US in the diagnosis of clinically symptomatic Achilles tendinopathy. A study by Klauser et al. [31] showed perfect agreement of CE with histology (Figs. 3 and 4), along with moderate agreement between conventional US and histology.

The use of SWE revealed high specificity [12, 53] in the detection of tendinopathy and the combined use of conventional US is recommended to increase the sensitivity of the diagnosis [52]. Coombes et al. described that a low Achilles V_s_ is correlated with higher age, self-reported pain and disability over the preceding week, and with decreased loading capacity of the Achilles tendon [53]. Studies in athletes demonstrated that tendon softening may predict pain and tendinopathy [38, 69–71]. Using conventional US, Fredberg et al. identified athletes with the greatest risk of developing tendon disorders [72]. Hence, USE may be useful in the detection of subclinical tendinopathy, providing time for prophylactic procedures (reduction of the load and eccentric training, for example). Once rehabilitation is considered the standard treatment for restoring the original mechanical properties of the tendon, it is crucial to assess the relation between clinical functional outcomes and the mechanical properties of the Achilles tendon.

In case of a tendon rupture, loss of tension, haematoma or effusion contribute to a significant decrease in stiffness, as verified by Chen et al. [54] and Aubry et al. [12], who identified signal-void areas at SWE. Domenichini et al. state that dynamic SWE is recommended for rupture diagnosis, since a totally ruptured tendon will not become taut when stretched [73].

USE was studied after the surgical repair of Achilles tendon rupture [17, 29, 30, 55]. In this scenario, there is an increase in tendon stiffness and heterogeneity according to the physiological healing process, probably due to the structural disorganisation of collagen fibres, with predominance of type III collagen instead of type I as usual. An increased stiffness in the contralateral tendon was also reported [74, 75] and that can be due to overload during the rehabilitation period or a sign of predisposition to tendinopathy [17]; however, the explanation remains unclear. Using SWE, Zhang et al. showed that, in addition to increased tendon stiffness, there was a positive correlation between the degree of tendon functionality and its elasticity, suggesting that lower elastic modulus values might predict poor mechanical properties, and functional outcomes, with worse healing of repaired tendons [55]. It is known that a repaired Achilles tendon never regains its normal US appearance. Therefore, USE is useful for monitoring tendon repair and rehabilitation, eventually avoiding re-rupture from early tendon loading [76].

Gehmert et al. carried out a laboratory study in rabbits demonstrating, through USE evaluation, that the application of mesenchymal stem cells in a ruptured Achilles tendon can totally restore their elastic properties [30]. Another recent laboratory study by Yamamoto et al. with Achilles transected tendons in rabbits described that, at the healing site, there was a gradual decrease of the strain ratio and the tendon became significantly harder over time [36]. They also demonstrated a correlation between the strain ratio and mechanical and histological properties of the healing tendon tissue. According to these findings, USE may reflect biological healing processes and, in that way, contribute to define the appropriate time to return to previous activity with lower risk of re-rupture.

USE has also been studied in systemic diseases. A stiffness reduction of the Achilles tendon was observed in patients with ankylosing spondylitis [32], in diabetic patients with foot ulcers [33] and in acromegaly patients [35].

### Patellar tendon

The reproducibility of the CE technique in healthy patellar was characterised with good intra- and inter-observer reliability by Porta et al. [77]. Excellent intra-observer reliability and moderate to excellent inter-observer reliability were reported for SWE [56, 68, 78].

Conventional US shows hypoechogenic regions in a tendon with patellar tendinopathy. However, asymptomatic individuals can have abnormal tendon morphology, including hypoechogenic regions at conventional US that do not help to predict the subsequent development of symptoms [79, 80].

Five studies using USE were found (two using CE and three using SWE) and the results were discrepant concerning elasticity characterisation. Three studies had results favouring a decrease in stiffness [37, 38, 52] and two studies, using SWE, described an increase of V_s_ and, therefore, stiffness in patellar tendinopathy [53, 56]. There are important methodological differences that can explain this disparity. It should be emphasised that different regions of the same tendon were assessed and different sizes of ROIs were used: Dirrichs et al. measured V_s_ using an ROI of 1 mm in diameter placed at the most rigid area of the tendon [52], Coombes et al. used a larger ROI and measured V_s_ at the middle and proximal parts of the tendon [53] and Zhang et al. [56] measured V_s_ at a proximal part. It has been shown that the elastic modulus is influenced by the size of the ROI (Kot et al. reported that the elastic modulus of the patellar tendon increased with increasing size of the ROI [81]), as well as by the assessed tendon portion [15, 77, 82].

The patellar tendon is connected to two hard and fixed structures (patellar bone and tibial tuberosity), contrary to the other studied tendons, which are connected on one side to a softer and more compliant structure (muscle). This fact is stated by Porta et al. as an explanation of their results, in which the healthy patellar tendon was characterised by a soft pattern using CE [77]. Therefore, it is not clear which pattern and elasticity values are able to differentiate a healthy patellar tendon from a patellar tendinopathy.

Zhang et al. assessed 13 athletes with unilateral patellar tendinopathy using SWE and found a correlation between increased stiffness of painful tendon and the intensity of pain and degree of dysfunction [56]. Using CE, Ooi et al. described symptomatic patellar tendons in athletes as softer than asymptomatic ones and better related to functional scores than conventional US. The same study showed that USE increased conventional US sensitivity and accuracy in the diagnosis of patellar tendinopathy [38].

Patellar tendinopathy is common among active athletes [83], so USE may be employed to assist sports medicine clinicians, providing a more effective rehabilitation in athletes with patellar tendinopathy.

### Epicondylar tendons

**Fig. 5:**
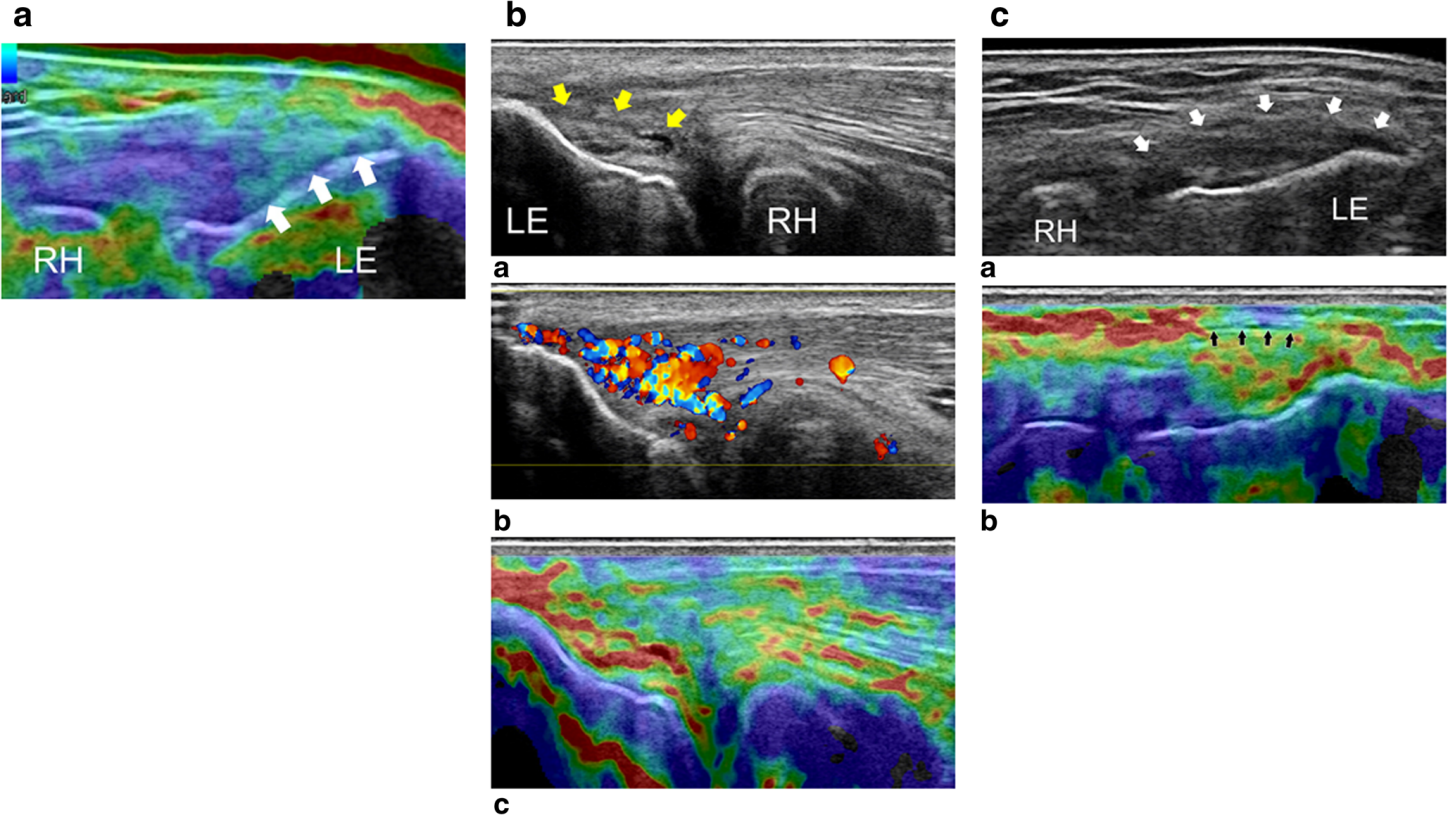
CE in the evaluation of the common tendon of the lateral epicondyle. **a** USE image of the common extensor tendon in the longitudinal plane in a healthy individual. The tendon insertion is shown to have a rigid homogeneous structure (arrows). **b** (*a*) The B-mode conventional US image of the extensor tendon in the longitudinal plane. High-grade tendinosis is seen with fibre dehiscence (yellow arrows). At the same level, Doppler (*b*) demonstrates hypervascularisation and the elastogram (*c*) shows pathological decrease of the common extensor tendon stiffness. **c** (*a*) B-mode conventional US image of the common extensor tendon in the longitudinal plane demonstrating bulging and thickening of the insertion (white arrows). At the same level, the elastogram (*b*) shows irregular stiffness, involving the peritendinous fascia (black arrows), with pathological adhesion between the tendon and the surrounding tissue. LE: lateral epicondyle, RH: radiohead. Reproduced, with permission, from Klauser et al. [26], copyright (2014) by the Radiological Society of North America, Inc. (RSNA)

Repetitive microtrauma is considered the main factor that leads to epicondylar tendinopathy. In lateral epicondylitis, extensor carpi radialis brevis (ECRB) is the most commonly affected muscle, but supinator and other wrist extensors can also be involved. Any activity involving excessive and repetitive use of these muscles can result in multiple microtears, leading to a cycle of tendon degeneration and repair [84–86]. The use of conventional US to differentiate the tissue affected by degenerative disease from the healthy tissue can be challenging because, often, both have the same echogenicity [86, 87]. Seven studies (six using CE and one using SWE) showed a decrease in stiffness pattern on symptomatic tendons [39–44]. For instance, Fig. 5 represents an elastogram and US images of a normal and a pathologic epicondylar tendon. The focal hypoechoic area in the deep part of the ECRB tendon, corresponding to collagen degeneration with fibroblastic proliferation, is the most common US finding of lateral epicondylitis [88]. Using CE on lateral epicondylitis, De Zordo et al. found slightly more focal lesions and a more frequent involvement of the lateral collateral ligament and the peritendinous fascia compared to conventional US [39]. Lateral collateral ligament tear and large intra-substance tears are related to poor outcomes when using non-operative treatment. In these cases, autologous blood injection, platelet-rich plasma cell therapy and surgery are approaches to be considered [89].

Park et al. described that USE findings were related to pain during resistive middle finger extension on physical examination, meaning that the superficial group was affected, and, therefore, the combination with conventional US may add information to differentiate the injured group fibres [41].

Three studies concluded that USE was superior to conventional US in terms of accuracy and/or sensitivity [39, 41, 42]. Klauser et al. studied epicondylar tendons in cadavers and concluded that the combination of USE and conventional US provided better correlation with histology than the use of both modalities alone [43, 44].

Correlations of USE findings with clinical symptoms scores [52], symptom duration and history of corticoid injection were found [41]. Studies addressing elasticity changes on follow-up and rehabilitation treatments were not found for epicondylar tendinopathy. The functional outcomes and the relation with USE should be further evaluated in future research.

### Rotator cuff tendons

In our research, five studies used CE and seven studies used SWE in cases of rotator cuff tendinopathy or supraspinatus tendon tear. Good to very good intra- and inter-observer repeatability were reported for measurement of V_s_ in the supraspinatus muscle and tendon [58, 90].

**Fig. 6:**
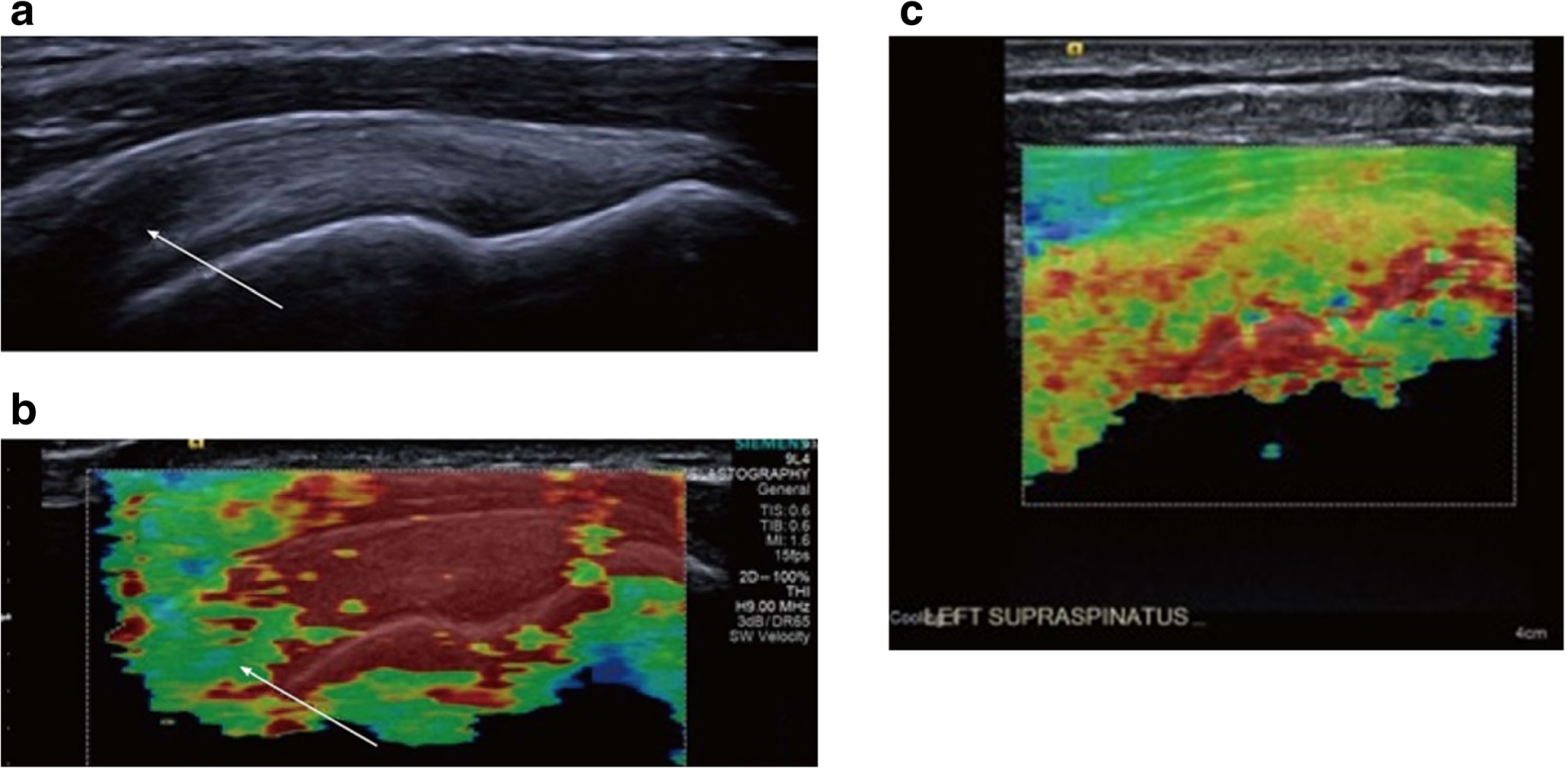
Images of supraspinatus tendon using SWE. **a** Appearance of a normal supraspinatus tendon showing anisotropy (white arrow) due to curvilinear orientation of the tendon. **b** Corresponding elastogram showing heterogeneous stiffness in the region of anisotropy and absence of measurement in the deepest region in the humeral head (which is expected since it is a high stiffness structure with limited propagation of the shear waves). **c** Longitudinal elastography by shear waves of the supraspinatus tendon with tendinopathy. An elastographic pattern of disorganisation and heterogeneity is evidenced, in contrast to the more homogenous pattern of a normal tendon (**b**). Adapted, with permission, from Winn et al. [18], copyright (2016) The Author(s). Published by Baishideng Publishing Group Inc. All rights reserved

Globally, a stiffness decrease and a heterogeneous pattern of softness are described in affected tendons compared to normal ones (Fig. 6) [46–48, 58, 60, 91]. This makes USE particularly interesting when conventional US cannot differentiate between healthy and pathological tissue. The findings on USE were correlated to magnetic resonance imaging (MRI) [45, 46, 49, 62] and conventional US [45, 46, 60]. Rosskopf et al. demonstrated that V_s_ decreased with increasing fat content and supraspinatus muscle atrophy, and was increased in the final stage of fatty infiltration [58]. Krepkin et al. found that V_s_ was negatively correlated with T2* weighted values measured on MRI sequences and tear size of the degenerated supraspinatus tendon, reflecting the tendon quality [62]. Seo et al. also stated that CE was able to quantify the severity of the fat atrophy of the supraspinatus tendon, reporting excellent accuracy and inter-observer reliability [45]. Since supraspinatus fatty infiltration and muscle atrophy can predict repeated tears rates and poor functional outcomes, it must be acknowledged that USE may confer prognostic utility in surgical repair decision [92, 93]. Hatta et al. found that quantitative SWE assessment of the supraspinatus muscle was highly correlated with extensibility of the musculotendinous unit on cadaveric shoulders. Therefore, USE could offer a non-invasive method to predict rotator cuff extensibility during the preoperative planning phase for a rotator cuff repair [61]. Hou et al. also hypothesised that SWE may be useful in the preoperative setting for patient selection and surgical planning, as it can reflect tendon quality or post-operative failure rates [60].

Muscle stiffness can be related to rotator cuff tendinopathy. An increased stiffness on the upper trapezius muscle assessed with SWE was associated with rotator cuff tendinopathy in a study of volleyball players by Lin et al. [57]. These authors suggested that athletes with increased shear modulus on the upper trapezius may have higher risk of developing rotator cuff tendinopathy and, consequently, USE applied in the upper trapezius can be employed for prevention/rehabilitation purposes. Another study found that deltoid muscle softening had a correlation with tendinopathy severity assessed by conventional US [60].

The evaluation of 39 patients with calcifying tendinopathy using SWE allowed to define a non-dark pattern that was a predictor of symptomatic relief after fine needle aspiration. These results reflect the utility of USE in the evaluation and management of rotator cuff calcific tendinosis [57].

### Other tendons

Tendinopathy of the long head of biceps tendon was characterised with CE in one study. The authors found that the affected tendons had softening areas on USE, with a positive correlation with conventional US [50].

The quadriceps tendon was also characterised with CE in patients undergoing chronic haemodialysis and it was described as being thinner and softer with greater colour heterogeneity on the elastogram compared to the controls [51].

## Limitations and recommendations

Despite the great interest in USE, the published literature is still very scarce and deeply focused on cross-sectional studies, not controlled and with small populations. USE techniques have several limitations that can affect the reproducibility and comparison of results.

CE is not a quantitative technique and, thus, has led to the use of alternative methods, such as the relative ratio of strain, different scoring and graduation systems, and external software [16, 64, 94, 95].

The elastogram represents relative strains, i.e. the elasticity of the structure under analysis is compared with that of the adjacent tissues, which can lead to great dispersion of the results. An acoustic coupler with a known Young’s modulus has been developed for a more consistent strain ratio measurement reference and was shown to be reproducible and to correlate with qualitative elastography measurements [65, 96, 97].

The window size may affect the stress distributions of the elastogram and should be standardised. A suggested standard size using CE states a depth of three times the tendon size and about three-quarters of the screen in longitudinal scans with the inclusion of the paratenon in transverse scans [95]. Kot et al. showed significant differences in the maximum elastic modulus for different sizes of ROIs in patellar tendon and rectus femoris muscle, proportionally increasing with the sizes of the ROI, possibly by including more muscle fascia and dense collagen fibre than in smaller ROIs [81].

Compression elastography is a highly operator-dependent technique and particularly vulnerable to intra- and inter-operator variability [64, 95, 98, 99]. The application of compression–decompression cycles with moderate and appropriate compressive force can be challenging. The application of too much or too little force affects the calculation of the strain due to non-linear elastic properties of the tissues [23]. Currently, several techniques and software integrate USE systems in order to provide visual feedback that ensures the correct application of pressure, helping to reduce inter-observer variability and assist image acquisition [64]. Several trials of compression cycles must be performed and the images should be obtained in the central phase of the compression, as they provide the best contrast. The images obtained at the beginning and at the end of each cycle are often inaccurate [16, 27].

Tissue slip artefacts occur when the ROI leaves the frame during the compression–decompression cycles. Such artefacts can be minimised by aligning the probe head with the region of interest during compression [18].

During the evaluation of structures with prominent bone projections, it is difficult to apply uniform compression, compromising the validity of the obtained elastograms. It should also be noted that cross-sectional elastograms, due to the higher probability of artefacts, are inferior in quality compared to longitudinal perspectives [64, 100]. In addition, persistent fluctuations in the distribution of stresses during compression occur at the limits of the longitudinal elastogram. To exclude results in these limits, overlapping elastograms must be obtained. For example, dividing the Achilles tendon into three parts allows the overlap of elastograms to not include the limits [101].

SWE allows a more objective data quantification and is more reproducible; however, most of the commercial US devices do not provide this modality yet [9, 102].

Most US equipment with elastography requires a minimum distance (usually 1–2 mm) between the structure of interest and the surface of the skin for the calculation of the elastograms. In lean individuals, this minimum distance may not be observed, leading to the use of gel spacers to meet this requirement, which is not proved to accurately produce the same results [16, 27, 64, 95]. Transducer pressure affects not only CE measurements but also SWE parameters. Kot et al. reported that the measured elastic modulus of the rectus femoris and the patellar increased with added transducer pressure [81]. Conversely to CE, SWE does not require deformation or compression of targeted tissues and, for that reason, light pressure with the transducer is recommended.

The disposition of the structure to be examined as well as the probe position will also influence the results obtained with USE. Two studies using USE in the patellar tendon reported that elasticity was greater in flexion and less in the full extension position [103, 104].

A comparative cross-sectional study between professional athletes and healthy volunteers concluded that using CE in the patellar portion of the tendon presents greater stiffness when compared to the tibial portion [82]. Several studies using SWE emphasised the importance of both spatial location and ankle posture in the evaluation of the Achilles tendon [12, 74, 105, 106]. Helfenstein-Didier et al. found that the Achilles tendon shear modulus increased with passive dorsiflexion and, regardless of the ankle angle, it was significantly higher in the proximal region of the tendon [102]. Aubry et al. also confirmed that stiffness of the Achilles tendon increased when it was stretched, and that V_s_ values were higher when the measurements were acquired parallel to the fibres as opposed to perpendicular to the fibres, because of tendon anisotropy [107]. The highly anisotropic nature of the tendon requires the positioning of the US probe in a perfectly parallel or perpendicular direction to the fibres [108]. The abduction angle of the shoulder may affect USE results when assessing the supraspinatus tendon. Baumer et al. found lower mean V_s_ for the supraspinatus muscle and tendon compared to the mean V_s_ reported by Rosskopf et al., suggesting that V_s_ values may decrease with increasing abduction angle [58, 63].

The currently available USE systems are based on the pre-requisite assumption that tissue is homogenous and isotropic [109, 110], which is not completely true regarding tendons. The vendor system can be a source of variability and, for that reason, the same equipment, when following up patients, should be used to ensure reproducibility of the results [111, 112].

Ageing seems to be associated with poorer elastic properties of tendons, supported by USE usage in the assessment of the Achilles tendon, the patellar tendon and the supraspinatus muscle and tendon [15, 53, 63, 74, 106, 107, 113]. Microstructural changes including increased collagen cross-linking may be an explanation for the increase in tendon stiffness with ageing [114].

The stiffness of the tendon is also affected by mechanical loading and exercise [115–118]. Three studies using SWE reported that exercise is associated with greater elasticity of the Achilles tendon [119–121]. Furthermore, a significant correlation was found between CE results in the Achilles tendon and BMI. Another study found a negative correlation between patellar tendon thickness and strain ratio with smoking load [59, 122]. Therefore, it is important to consider that these factors may interfere with elastic properties on tendons and, consequently, limit the comparison between studies that used USE in different populations.

In what concerns the set of limitations related with USE, it is necessary to standardise the following: technical parameters (pressure applied to the probe, size of the region of interest, acquisition time, equipment used), examination protocols (including spatial arrangement of the probe and the structure to be analysed, as well as segmentation of the tendon portions), variables and classification systems. In general, it is imperative to guarantee the adequate reproducibility and comparability of the results.

Given the scarcity and limitations of the existing literature, a more structured and systematic approach is needed in the future with controlled multicentre studies, encompassing broader populations at different ages and with different activities and long-term follow-up. Studies that correlate with conventional imaging methods (MRI and US), histology and biomechanical and clinical data should be performed. Based on the available techniques of elastography, their role and importance also need to be assessed.

## Conclusions

Ultrasound elastography (USE) is a non-invasive technology that has evolved in recent years. This technology allows a qualitative and quantitative evaluation of the mechanical properties of the tissues adjuvant to conventional ultrasound (US).

Shear-wave elastography (SWE) is considered more objective, quantitative and reproducible than compression elastography (CE).

USE also confers potential value to conventional US due to increased sensibility and accuracy in the evaluation of tendinopathies. Studies to date suggest that elastic properties of tendons change under pathological conditions, identifiable with USE even before they are visible with conventional imaging methods. USE may be useful in early diagnosis, delineation of the extent and degree of tendinous degeneration, tracking outcomes of treatments, including the post-surgical follow-up, and as a screening tool in the sports context, allowing athletes to modify their training plan to avoid tendon injury. It could also provide insights for physical medicine and rehabilitation researchers in what concerns tendon properties and their impact on function.

Currently, USE presents several limitations, being highly subjective, lacking standardisation, affecting the inter-operator variability and reproducibility of the results obtained. The applicability in the evaluation of tendon injury is not yet perfectly defined, and SWE seems to better respond to some of the limitations compared to CE, because it provides more objective results and less technical variations. It is our opinion that the use of USE should be reserved for specialised centres at the present time to serve diagnosis, monitoring and research purposes, where the results can be compared with other imaging techniques and integrated into clinical practice by multidisciplinary teams.

Thus, an appropriate standardisation and structured future research are required in order to reinforce the role of USE as a valuable complementary technique in the assessment of tendon injury for diagnostic purposes, treatment decisions and response evaluation to rehabilitation interventions.

## Abbreviations

CE: Compression elastography
SWE: Shear-wave elastography
US: Ultrasound
USE: Ultrasound elastography
